# Medicinal Treatment of Irritable Temper

**Published:** 1896-11

**Authors:** 


**Medicinal Treatment of Irritable Temper.**—In the September number of the *Glasgow Medical Journal* there is an abstract of an article from the July number of the *Practitioner*, in which the writer says that Dr. Lauder Brunton has paid some attention to the subject of bad temper as an indication of diseased conditions, and to the method by which relief of a symptom so personally and socially distressing can be obtained. Some time ago he noted that unwonted irritability of temper was often the precursor of a headache, and described the beneficial action of bromide of potassium and salicylate of sodium in relieving the headache. He now recommends the same combination for irritability of temper occurring in connection with various diseases, and more especially in gout and heart disease. The beneficial effect of the bromide upon the irritable nerve centres is, of course, universally recognized, and Dr. Brunton considers that the researches of Dr. Alexander Haig justify the conclusion that the salicylate of sodium is of value by promoting the elimination of uric acid. Referring to irritability of temper as a symptom of cardiac disease, Dr. Brunton remarks upon its frequency, and quotes the case of a child in whom it was the only symptom of mitral regurgitation, the physical evidence of the disease being observed almost by accident. He finds the above-mentioned remedies to be valuable adjuncts to the use of digitalis and other cardiac tonics. They improve the subjective condition of the patient, and thus facilitate his recognition of improvement.—*N. Y. Med. Journal.*

